# Supplementary material for: Suicide adverse events associated with zopiclone and eszopiclone: A pharmacovigilance analysis based on FAERS, JADER and CVARD
Source: PLoS One. 2026 Jan 8;21(1):e0340357. doi: 10.1371/journal.pone.0340357 (PMC12782430; doi:10.1371/journal.pone.0340357)
Supplement: S1 Table — (DOCX) [file pone.0340357.s001.docx]

Supplementary Table 1:

Two-by-two contingency table for disproportionality analyses.

|  | AEs of suicide | All other AEs | Total |
| --- | --- | --- | --- |
| Zopiclone and Eszopiclone | a | b | a+b |
| All other drugs | c | d | c+d |
| Total | a+c | b+d | a+b+c+d |

Abbreviation: AEs, adverse events; a, number of reports containing both the zopiclone and eszopiclone and AEs of suicide; b, number of reports containing other adverse drug events of the opiclone and eszopiclone; c, number of reports containing the AEs of suicide of other drugs; d, number of reports containing other drugs and other adverse drug events.
