# Supplementary material for: Suicide adverse events associated with zopiclone and eszopiclone: A pharmacovigilance analysis based on FAERS, JADER and CVARD
Source: PLoS One. 2026 Jan 8;21(1):e0340357. doi: 10.1371/journal.pone.0340357 (PMC12782430; doi:10.1371/journal.pone.0340357)
Supplement: S2 Table — (DOCX) [file pone.0340357.s002.docx]

Supplementary Table 2:

Four major algorithms used for signal detection.

| Algorithms | Equation | Criteria |
| --- | --- | --- |
| ROR | ROR=ad/b/c | lower limit of 95% CI>1, N≥3 |
|  | 95%CI=e^ln(ROR)±1.96(1/a+1/b+1/c+1/d)^0.5^ |  |
| PRR | PRR=a(c+d)/c/(a+b) | PRR≥2, χ^2^≥4, N≥3 |
|  | χ^2^=[(ad-bc)^2](a+b+c+d)/[(a+b)(c+d)(a+c)(b+d)] |  |
| IC | IC=log_2_a(a+b+c+d)(a+c)(a+b) | IC025>0 |
|  | 95%CI= E(IC) ± 2V(IC)^0.5 |  |

Abbreviation: a, number of reports containing both the zopiclone and eszopiclone and AEs of suicide; b, number of reports containing other adverse drug events of the opiclone and eszopiclone; c, number of reports containing the AEs of suicide of other drugs; d, number of reports containing other drugs and other adverse drug events. 95%CI, 95% confidence interval; N, the number of reports; χ2, chi-squared; IC, information component; IC025, the lower limit of 95% CI of the IC; E(IC), the IC expectations; V(IC), the variance of IC.
