# Supplementary figures and images for: Suicide adverse events associated with zopiclone and eszopiclone: A pharmacovigilance analysis based on FAERS, JADER and CVARD
Source: PLoS One. 2026 Jan 8;21(1):e0340357. doi: 10.1371/journal.pone.0340357 (PMC12782430; doi:10.1371/journal.pone.0340357)

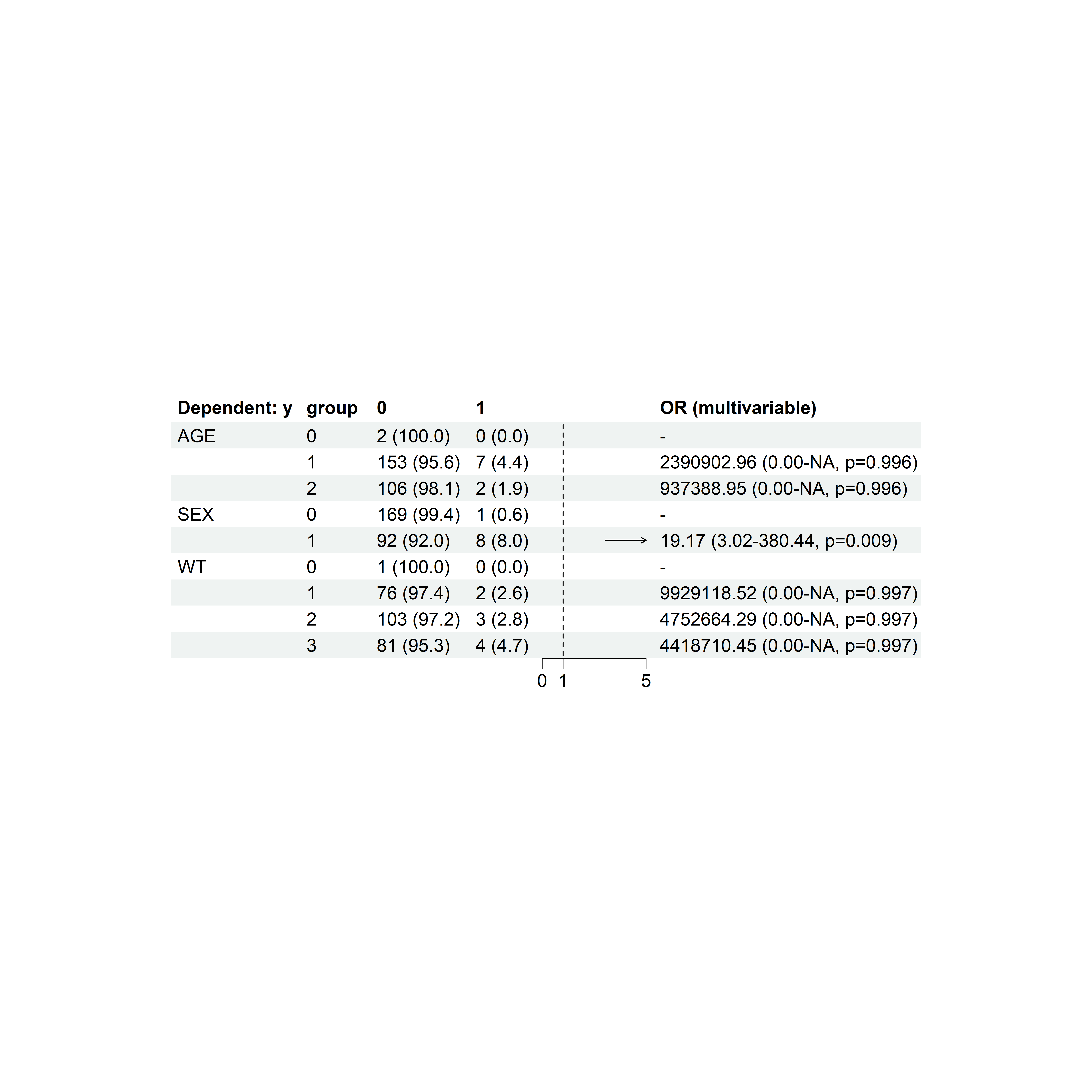

Supplement: S3 File — (ZIP) [file pone.0340357.s003.zip › The data involved in the research--Revised-- 12.24/Logistic regression data/Final_logistic_Multivariate-forest map.png]

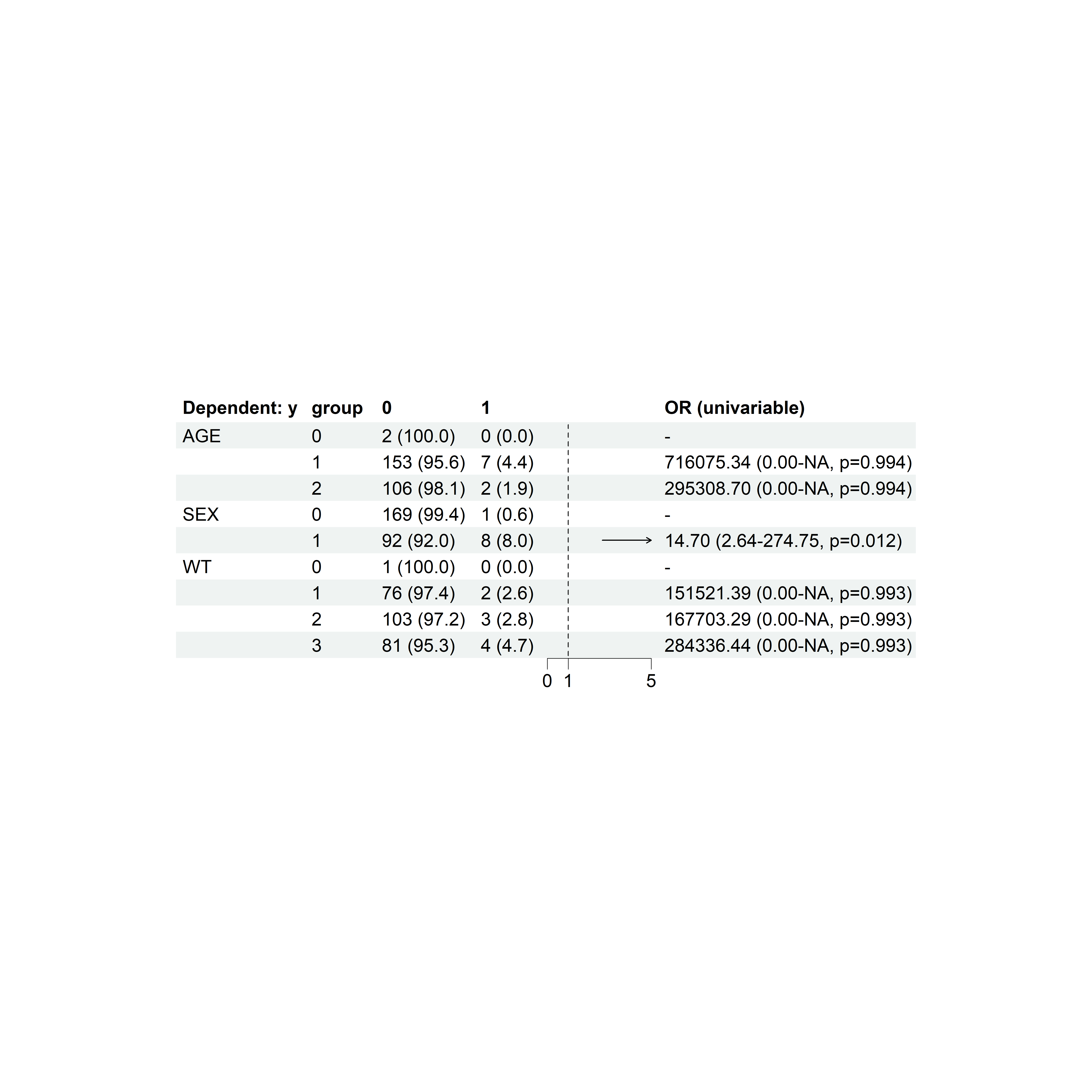

Supplement: S3 File — (ZIP) [file pone.0340357.s003.zip › The data involved in the research--Revised-- 12.24/Logistic regression data/Univariate_logistic_Results-forest map.png]

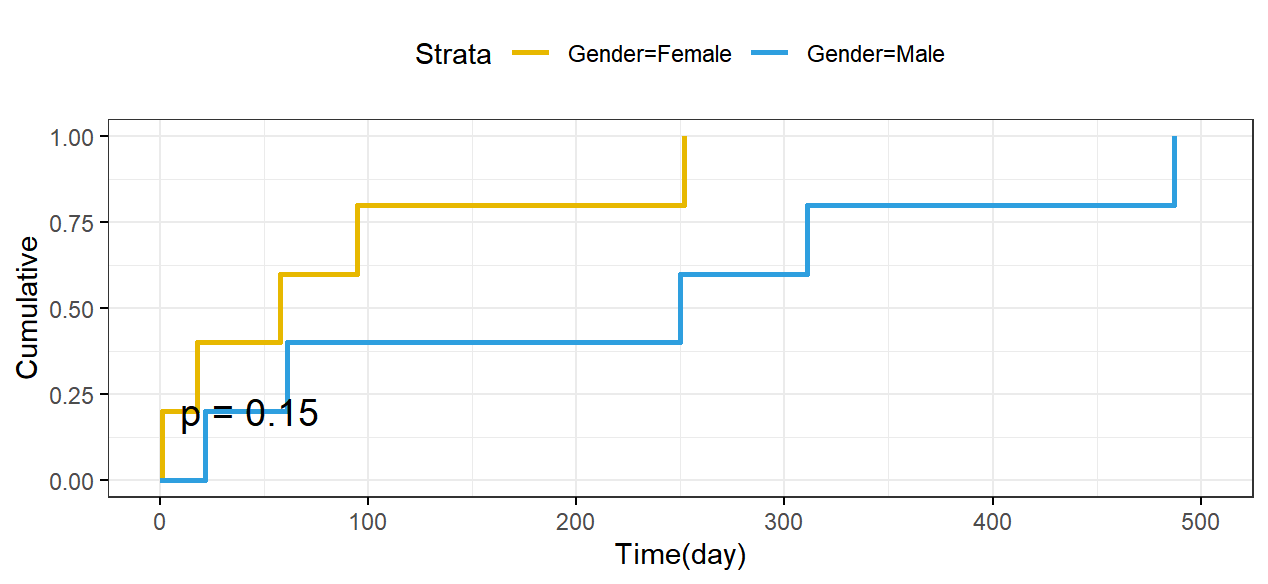

Supplement: S3 File — (ZIP) [file pone.0340357.s003.zip › The data involved in the research--Revised-- 12.24/Survival curve graph of adverse reaction induction time/FAERS.png]

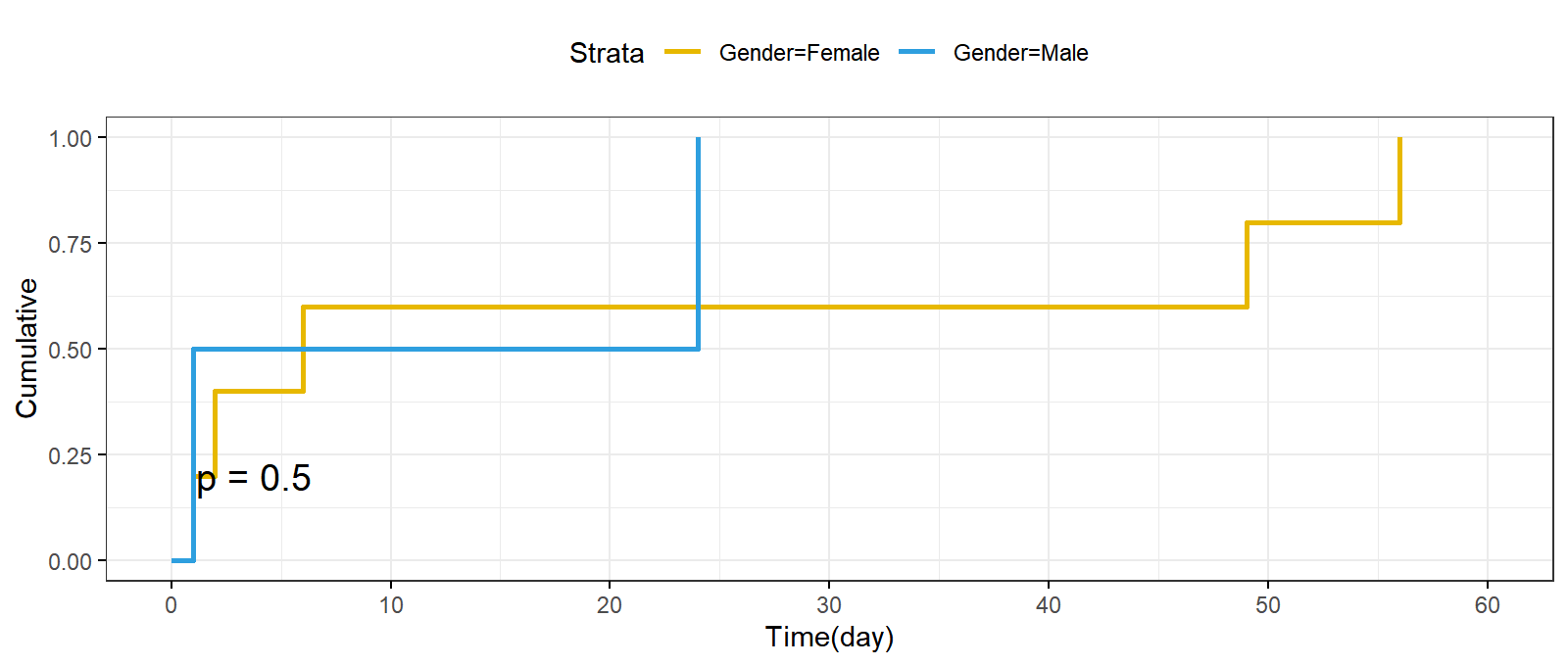

Supplement: S3 File — (ZIP) [file pone.0340357.s003.zip › The data involved in the research--Revised-- 12.24/Survival curve graph of adverse reaction induction time/JADER.png]

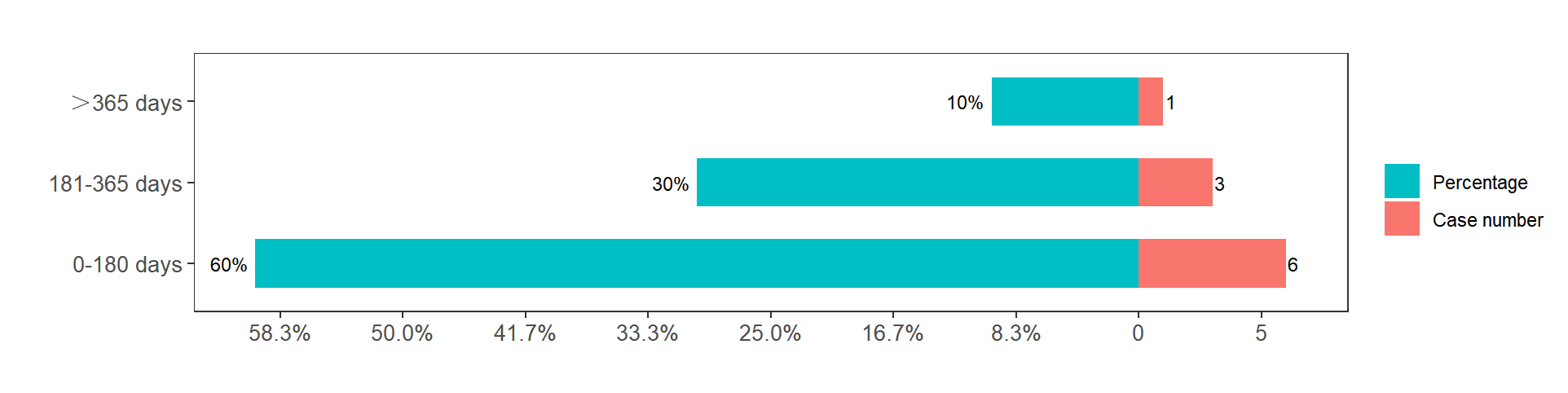

Supplement: S3 File — (ZIP) [file pone.0340357.s003.zip › The data involved in the research--Revised-- 12.24/TTO drawing data/Rplot.png]

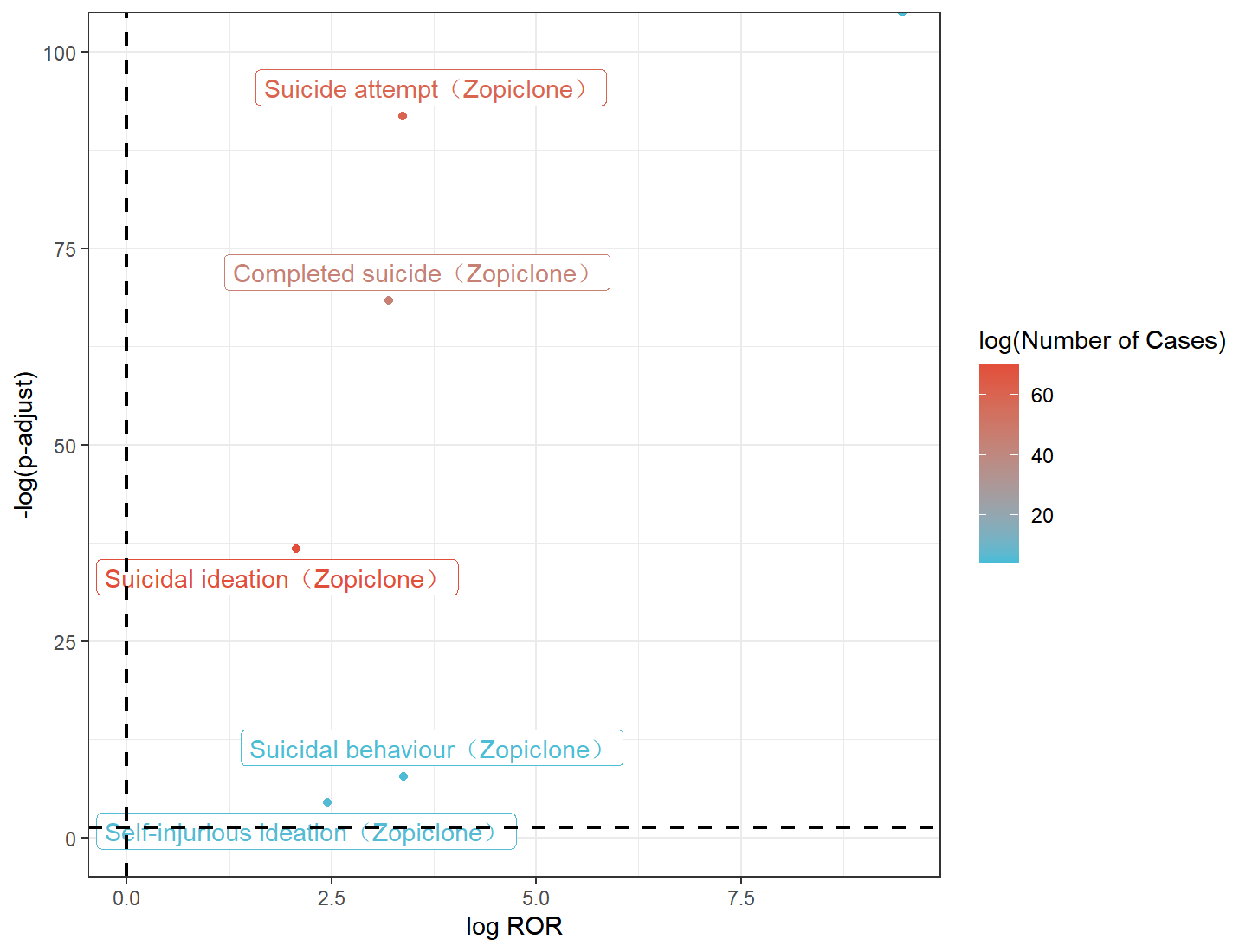

Supplement: S3 File — (ZIP) [file pone.0340357.s003.zip › The data involved in the research--Revised-- 12.24/Volcano map/CAVRD.png]

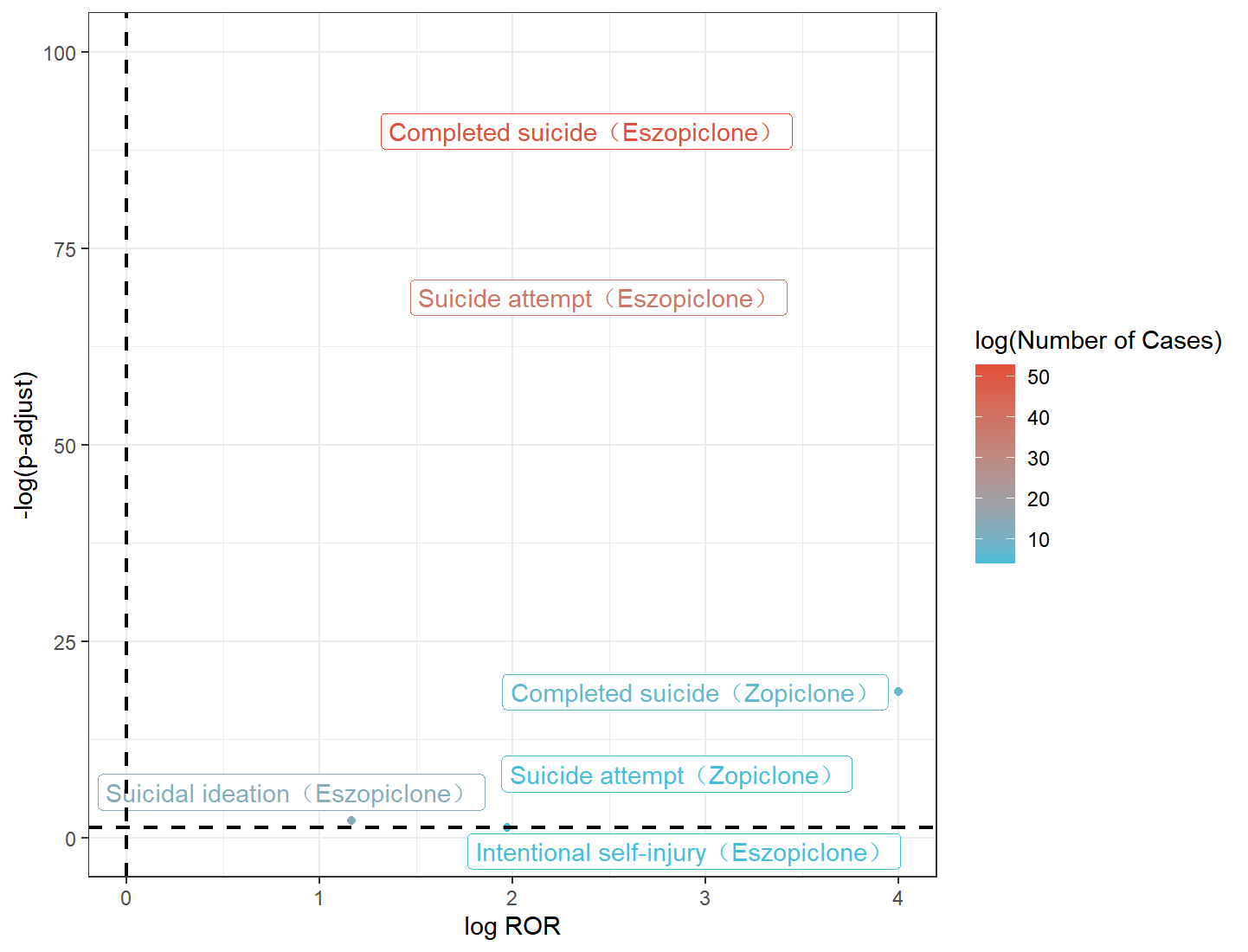

Supplement: S3 File — (ZIP) [file pone.0340357.s003.zip › The data involved in the research--Revised-- 12.24/Volcano map/FAERS.png]

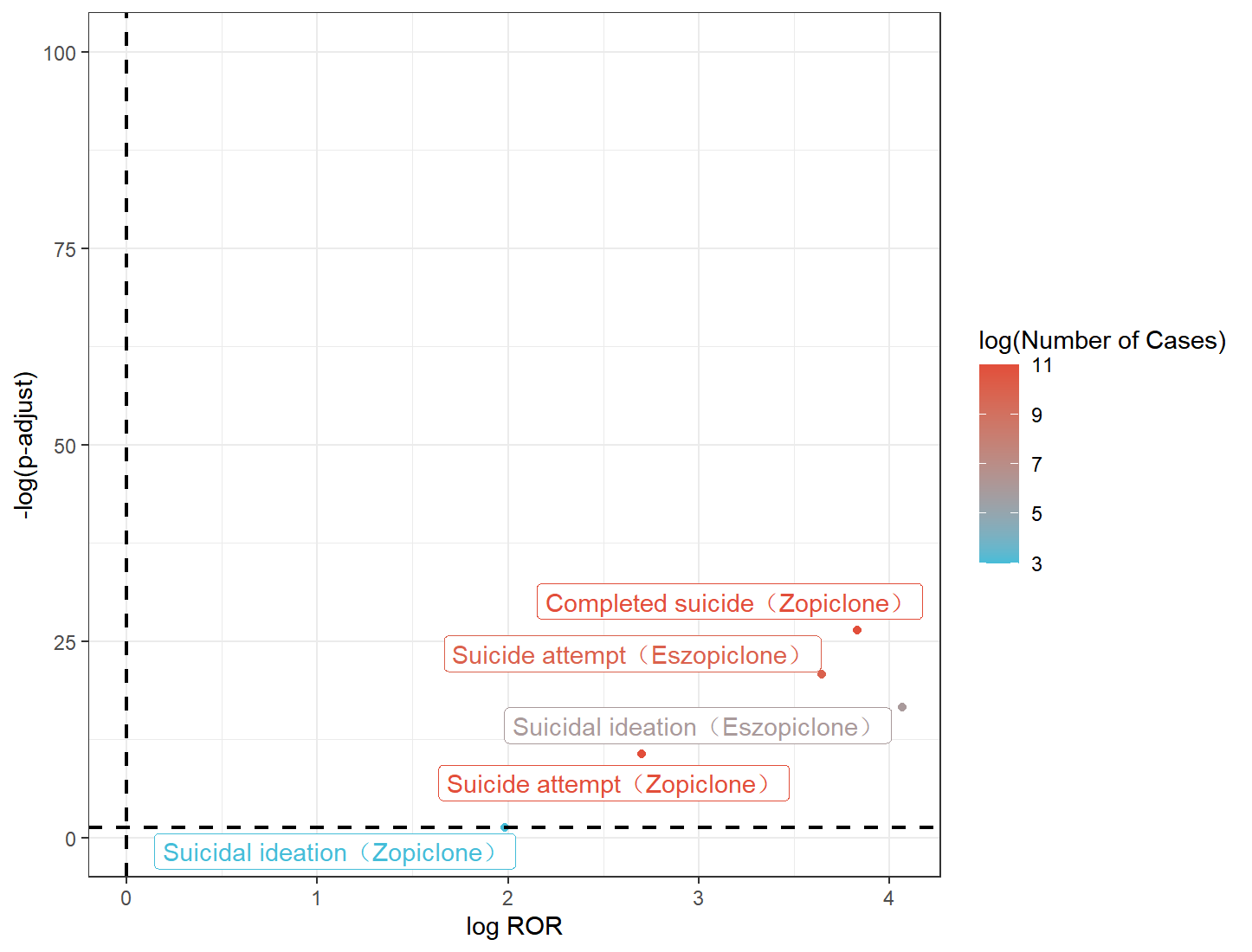

Supplement: S3 File — (ZIP) [file pone.0340357.s003.zip › The data involved in the research--Revised-- 12.24/Volcano map/JADER.png]
